# Attempted Training of Alcohol Approach and Drinking Identity Associations in US Undergraduate Drinkers: Null Results from Two Studies

**DOI:** 10.1371/journal.pone.0134642

**Published:** 2015-08-04

**Authors:** Kristen P. Lindgren, Reinout W. Wiers, Bethany A. Teachman, Melissa L. Gasser, Erin C. Westgate, Janna Cousijn, Matthew C. Enkema, Clayton Neighbors

**Affiliations:** 1 Department of Psychiatry, University of Washington, Seattle, Washington, United States of America; 2 ADAPT-lab, Department of Developmental Psychology, University of Amsterdam, Amsterdam, The Netherlands; 3 Department of Psychology, University of Virginia, Charlottesville, Virginia, United States of America; 4 Consortium Individual Development, Department of Developmental and Experimental Psychology, Utrecht University, Utrecht, The Netherlands; 5 Department of Psychology, University of Washington, Seattle, Washington, United States of America; 6 Department of Psychology, University of Houston, Houston, Texas, United States of America; University of Ariel, ISRAEL

## Abstract

There is preliminary evidence that approach avoid training can shift implicit alcohol associations and improve treatment outcomes. We sought to replicate and extend those findings in US undergraduate social drinkers (Study 1) and at-risk drinkers (Study 2). Three adaptations of the approach avoid task (AAT) were tested. The first adaptation – the approach avoid training – was a replication and targeted implicit alcohol approach associations. The remaining two adaptations – the general identity and personalized identity trainings – targeted implicit drinking identity associations, which are robust predictors of hazardous drinking in US undergraduates. Study 1 included 300 undergraduate social drinkers. They were randomly assigned to real or sham training conditions for one of the three training adaptations, and completed two training sessions, spaced one week apart. Study 2 included 288 undergraduates at risk for alcohol use disorders. The same training procedures were used, but the two training sessions occurred within a single week. Results were not as expected. Across both studies, the approach avoid training yielded no evidence of training effects on implicit alcohol associations or alcohol outcomes. The general identity training also yielded no evidence of training effects on implicit alcohol associations or alcohol outcomes with one exception; individuals who completed real training demonstrated no changes in drinking refusal self-efficacy whereas individuals who completed sham training had reductions in self-efficacy. Finally, across both studies, the personalized identity training yielded no evidence of training effects on implicit alcohol associations or alcohol outcomes. Despite having relatively large samples and using a well-validated training task, study results indicated all three training adaptations were ineffective at this dose in US undergraduates. These findings are important because training studies are costly and labor-intensive. Future research may benefit from focusing on more severe populations, pairing training with other interventions, increasing training dose, and increasing gamification of training tasks.

## Introduction

The implicit tendency to approach rather than avoid alcohol (i.e., implicit alcohol approach bias) is thought to play an important role in the course of alcohol use and dependence [1]. Moreover, approach bias retraining shows promise as an adjunct to standard addiction treatments [2,3]. To date, published research on approach bias retraining has largely focused on European samples of alcohol-dependent patients [2,3], targeting approach and avoidance responses towards impersonal alcohol-related images. The current studies were conducted to extend this important line of research in two ways. First, we sought to replicate previous approach bias retraining studies from Europe using US samples of heavy drinking young adults. Much of the research on implicit alcohol approach bias was conducted on this population [4–7], and there is considerable concern about the risks of and the harm from drinking that this group experiences [8], but training has not yet been evaluated with this group. Second, we sought to adapt approach bias retraining tasks to target a different implicit tendency, the tendency to associate one’s self with drinking alcohol (i.e., implicit drinking identity), because this tendency has recently been found to be a robust, consistent predictor of hazardous drinking in the US [4,5,9].

### Why Alcohol Approach Bias Retraining?

The interest in retraining alcohol approach bias is related to theoretical models in psychology and alcohol research that are commonly referred to as dual process models of cognition [1,10–12]. These models focus on two types of cognitive processes: (1) slower/deliberate/reflective (explicit) cognitive processes that are typically assessed by self-report questionnaires; and (2) faster/impulsive/reflexive (implicit) cognitive processes that are commonly evaluated using computer-based tasks that measure reaction time classifying stimuli to assess implicit associations. Findings from studies evaluating both kinds of processes indicate that both predict unique variance in behavior, including drinking and other maladaptive behaviors, and psychopathology [13–15]. With respect to implicit alcohol approach bias, specifically, there are numerous findings using multiple methods that indicate that stronger implicit associations to approach alcohol are associated with more hazardous drinking and that they predict unique variance in critical drinking outcomes, even after controlling for self-report questionnaires [4,7,14].

As the field has advanced, attention has turned to ways that implicit alcohol approach biases could be modified to reduce drinking. There is preliminary evidence for the efficacy of training tasks to modify associations [1,3], and drinking and treatment outcomes [2,3]. Training is not only exciting because it targets a unique risk factor for alcohol use disorders, but also because it may be a means to increase treatment access given it is computer-based. That is, it may be possible to develop computer- (and ultimately, Internet-) based interventions, which could increase access to treatment for individuals who currently lack access. This is critical: hazardous drinking and alcohol dependency have huge societal costs with tremendous global and national impact [16,17], yet the majority of those who could benefit from treatment do not receive it [18,19].

### Using the Alcohol Approach Avoidance Task (AAT) for Training

We focus here on implicit alcohol approach training because of its early successful results, and the clear theoretical and clinical importance of reducing the tendency to approach alcohol. Other training programs have been developed to modify different aspects of implicit cognitive processes related to alcohol, including those targeting an attentional bias to alcohol related-cues, with promising first effects [20,21], but they are beyond the scope of this paper. The first published study on implicit alcohol training was a single-session lab-based study with Dutch undergraduates [22]. This study used an adaption of the alcohol approach avoidance task (AAT), which requires participants to repeatedly and rapidly move a joystick away from pictures of alcohol and toward non-drinking stimuli (and vice-versa) and evaluates participants’ approach bias (i.e., are they faster approaching or avoiding alcohol-related stimuli). The training versions of the AAT are essentially the same with a critical exception: instead of a 50:50 split of pushing alcohol/pulling non-alcohol images and pulling alcohol/pushing non-alcohol images, there is a 90:10 split of pushing alcohol/pulling non-alcohol images and pulling alcohol/pushing non-alcohol (or vice-versa). In other words, in the training version of the AAT, participants over-practice pushing alcohol away and pulling non-alcohol images toward themselves (or vice-versa). Results from the first training study indicated that a single training session shifted implicit alcohol approach associations, and successful training was associated, among the heavier drinking students, with lower alcohol consumption in a subsequent alcohol taste test [22]. This training approach was then extended to individuals receiving inpatient treatment for alcoholism in Germany, and four AAT training sessions led to a reduction in implicit alcohol approach associations and even greater abstinence from alcohol one year later [3]. This finding was replicated in a subsequent study of alcohol-dependent patients who completed 12 sessions of training in addition to treatment as usual, which also provided evidence that the reduction in implicit alcohol approach associations was a mediator of the effect of training on treatment outcomes [2]. Collectively, findings from these studies suggest that training implicit alcohol approach associations can lead to clinically important outcomes. Moreover, the strength of the above findings encouraged us to extend this approach to young adults in the US.

#### Adapting the AAT to Target Implicit Drinking Identity

We also sought to adapt the AAT (and the AAT training paradigm) to target implicit drinking identity because having strong associations with the self and drinking alcohol has emerged as an important predictor of hazardous drinking in US young adults [4,5,9]. Furthermore, there is preliminary evidence that, among this group, implicit drinking identity associations may be even stronger predictors of drinking outcomes than approach avoid associations [4,5]. Consequently, in these studies, we used the original AAT and we developed and tested two adaptations. For the first adaptation, the “general identity” AAT, we changed the stimuli to target identity (i.e., the stimuli were words describing drinking alcohol or important, non-drinking aspects of identity for US undergraduates), and we used the same set of stimuli for all participants assigned to that adaptation. Because of the possibility that the general identity AAT might not adequately capture the aspects of identity most important to individual participants, a second adaptation was developed. This version, the “personalized identity AAT,” was identical to the general identity AAT with the exception that each participant selected the identity words used as stimuli.

### Purpose of the Present Studies

We had two goals. First, we sought to extend training to undergraduate drinkers in the US. Results from Wiers et al. [22] suggested that implicit alcohol associations can be shifted in Dutch undergraduates, and we were interested in evaluating whether those results would generalize to US populations. Further, the AAT training conditions used in Wiers et al.’s [22] included one condition that over-trained pushing alcohol away and another condition that over-trained pulling alcohol close. The study did not include a control training condition. Accordingly, we evaluated the AAT training condition that over-trained pushing alcohol away in comparison to a control condition (sham training) that was a continuation of the 50:50 AAT bias assessment (this strategy was used successfully in training studies with alcohol dependent patients, [2, 3]. We also increased the length of the training session, essentially giving participants a double-dose of training, with the goal of increasing the effectiveness of training. Our second goal was to adapt the AAT to target implicit drinking identity associations. We developed two adaptations for the drinking identity AAT: (1) a general drinking identity AAT that used a standardized set of stimuli that reflected important, non-drinking aspects of identity for undergraduates, and (2) a personalized identity AAT that allowed participants to select stimuli that reflected important, non-drinking aspects of their identity. Additional modifications to the AAT focused on clarifying the connection between pushing/pulling stimuli and the self.

### Overview of Studies

In Study 1, we evaluated and compared the effectiveness of three AAT training variants (alcohol approach, general drinking identity, personalized drinking identity) in a sample of undergraduates who reported at least one heavy drinking episode in the last month. We evaluated the effect of two sessions (spaced one week apart) of training on their implicit alcohol associations, future drinking intentions, and craving for alcohol. Across all three training approaches, we expected to find reductions in implicit alcohol associations, future drinking intentions, and craving for individuals assigned to the AAT training versus the control groups (sham training). In study 2, we evaluated the same AATs on a higher risk sample—undergraduates at risk for alcohol use disorders (i.e., with AUDIT scores of eight or more)–and participants completed two training sessions in a single week. We again expected to find reductions in implicit alcohol associations, future drinking intentions, and craving for individuals assigned to the AAT training versus the control group (sham training).

## Study 1

### Method

#### Participants

Participants (*N* = 295; 161 women, 133 men, 1 did not provide a response) were recruited from a large Pacific Northwest public university. They were undergraduates between the ages of 18 and 25 years (*M* = 20.48, *SD* = 1.42), fluent in English, and reported at least one heavy drinking episode (4/5 or more drinks for women/men) in the past month. Given the following choices of racial identity as outlined by the US National Institutes of Health, seventy percent characterized themselves as White, 21% Asian, 6% multiracial, 1% African American, and less than 1% each as Native Hawaiian or other Pacific Islander and American Indian/Alaska Native. The remaining 2% selected unknown or did not provide a response. Additionally, 5% identified their ethnicity as Hispanic or Latino, 94% as not Hispanic or Latino, and 1% as unknown.

#### Measures and Materials

The Alcohol AAT (Approach Avoidance Task)[23,24] is a computerized task in which participants push or pull stimuli on a computer screen using a joystick. As participants pull a stimulus, it grows bigger on the screen, and as participants push it, it shrinks. We used three variants of the AAT: the original AAT, the general identity AAT, and the personalized identity AAT. In the original AAT, participants viewed 10 images of alcoholic beverages and 10 images of water. Each image was presented in either a horizontal (landscape) or vertical (portrait) format. Participants were instructed to pull landscape-oriented images towards themselves using the joystick and to push portrait-oriented images away (push/pull image format instructions were counterbalanced across individuals). The AAT includes both a bias assessment and training phase, as outlined in previous work by Wiers et al. [3,22].

All participants completed an initial practice phase before beginning the bias assessment. During the 80-trial bias assessment, alcohol- and non-alcohol images were distributed equally in portrait- and landscape-formats, so that participants were asked to push alcohol images 50% of the time and pull alcohol images 50% of the time. The AAT then shifted from bias assessment to the training phase, which consisted of three blocks of 200 trials each (which is double the length of training sessions in previous studies), with a short break halfway through. During the break, participants could stand up and step away from the computer; they did not complete any other tasks or measures. For participants in the control condition (i.e., sham training), the AAT continued in the same 50–50 bias assessment format for those 600 trials. For participants in the experimental condition, the ratio of alcohol and non-alcohol images in the portrait and landscape-formats was altered so that participants were pushing alcohol images away 90% of the time and pulling non-alcohol images towards them 90% of the time.

The general identity AAT used the same procedure with two critical modifications. The first concerned what participants saw when they pushed or pulled stimuli. In the original AAT, images that are pushed get smaller on the screen (as if they zoom away) and images that pulled get larger (as if they zoom closer). In the adaptation, the images still zoom, but those that are pulled get larger and move closer to the word “Me” that appears on the bottom of the screen and those that are pushed get smaller and move closer to the word “Not me” on the top of the screen. The rationale behind this change was to clarify the connection between zooming away/closer and the self/not-self. The second modification concerned the AAT stimuli. In the original AAT, stimuli are images of alcohol or non-alcohol. In the general identity AAT, the stimuli were changed to target identity specifically. Thus, we used 20 words describing drinking alcohol (e.g., alcohol, beer, drinking, chugging, buzzed) and 20 words describing important, non-drinking aspects of identity (e.g., student, movies, working-out, friends, helpful). These words were presented in landscape or portrait formats. The non-drinker words were selected from online data from more than 1000 undergraduates who were initially screened for this study. They were asked to lists aspects of their identity that were not related to drinking and that were positive and important to them in five domains (athletics/recreation, hobbies/activities, academics/occupations/vocations, relationships, extracurricular/group membership). The domains and wording used were adapted from Oishi et al. [25] and Stryker and Serpe [26]. The four most frequent responses for each category were used for the non-drinker words. The drinking words were matched on part of speech and word length.

The personalized identity version used the same procedure. However, instead of using the same set of non-drinker words for all participants, each participant was asked to select words with which they strongly identified. Participants were presented with a list of 20 words from each of the five domains described above (they were the most frequent responses for each domain from the screening data). They selected four words from each domain for a total of 20. The selected words were subsequently used as the non-drinking stimuli for that participant. The drinking stimuli were the same items used in the general identity version.

The AAT bias assessment was scored using the D-score strategy (based on Greenwald et al. [27], and adapted for the AAT by Wiers et al [3]), which results in two scores—a bias score for alcohol-related stimuli and a bias score for non-alcohol-related stimuli, with higher scores indicating faster reaction time pulling stimuli toward one’s self. In addition, we calculated an overall AAT bias score using the same strategy used to score an IAT D score. Specifically, we subtracted the mean reaction time for alcohol pull and non-alcohol push trials from the mean reaction time for alcohol push and non-alcohol pull-trials and divided that by the standard deviation of all trials. Higher scores would indicate stronger alcohol-approach (or drinker-me) associations. Internal consistencies were calculated by computing the split half reliability of the overall AAT D-score for the 80-trial bias assessment (*r*s for overall D-score: original AAT = .00, general identity AAT = .06, personalized identity AAT = .02). Additionally, we computed the split half reliability for the alcohol bias D scores (original AAT = .16, general identity AAT = .07, personalized identity AAT = .30) and the non-alcohol bias D scores (original AAT = .27, general identity AAT = .41, personalized identity AAT = .12). Internal consistency was low.

The Implicit Association Test (IAT) [28] was used as an additional measure of implicit alcohol associations. It is a computerized reaction time task that assesses the relative strength of associations between two pairs of concepts. Participants use two keys on a keyboard to categorize target stimuli appearing one at a time on the screen as belonging to one of four categories (e.g., "Alcohol," "Water," "Approach," "Avoid"). The average response latencies in categorizing stimuli correctly are compared between two response pairing conditions. For instance, we can compare response latencies when A) *Alcohol* and *Approach* items are categorized with one response key (‘e’) and *Water* and *Avoid* items are categorized with the other (‘i’) with the pairing condition where B) *Alcohol* and *Avoid* items are categorized with one response key and *Water* and *Approach* items are categorized with the other. Participants who categorize items faster in the first condition compared to the second are said to have an implicit association between alcohol and approach tendencies. The IAT followed the standard 7-block format described in Greenwald et al. [28]. Order of the two category pairing conditions was randomized across participants. We computed scores using the *D* algorithm recommended by Greenwald and colleagues [27].

We assessed two different alcohol-related associations: alcohol approach and drinking identity (see Lindgren et al., 2013a). For the alcohol approach IAT, we used five images of alcohol (e.g., beer, liquor, wine) and five images of water. Approach and avoid were represented by words (e.g., approach, toward, avoid, leave). For the drinking identity IAT, drinker and non-drinker words (e.g., drinker, partier, abstainer, non-drinker) were paired with words representing the self or other (e.g., me, self, they, other). IATs were scored such that higher scores indicated stronger associations between alcohol-approach (and water-avoid) and drinker + me (and non-drinker + not-me), respectively. Each IAT’s internal consistency was calculated by computing two *D* score algorithms (one from the trials from third and sixth blocks and one from the trials from the fourth and seventh blocks) and then calculating the Pearson *r*, an approach used by Greenwald et al. (2003; alcohol approach *r* = .48, drinking identity *r* = .39). Internal consistencies for the drinking identity IAT were lower than observed in other studies with similar samples [5].

In the online screening survey, typical weekly alcohol consumption was calculated using the Daily Drinking Questionnaire (DDQ) [29]. Participants reported the number of drinks they consumed on each day of a typical week during the last three months using provided standard drink volume definitions (4 oz. wine, 12 oz. beer, 10 oz. microbrew beer, 1.5 oz 80-proof hard liquor). Cronbach’s alpha = .69.

Participants reported the number of heavy episodic drinking occurrences they had experienced in the last month, defined as four or more drinks on a single occasion for females, or five or more for males.

The Alcohol Use Disorders Identification Test (AUDIT) [30] contains 10 questions evaluating consumption, dependence and consequences related to alcohol. Elevated summed scores indicate a greater likelihood of disordered alcohol use. Cronbach’s alpha = .79.

The frequency of alcohol-related problems in the previous three months was assessed with the 25-item Rutgers Alcohol Problem Index (RAPI) [31]. Two items were added assessing the regularity with which participants drove shortly after having two or four drinks, respectively. All items were rated using a range of five options (“never” (0) to “more than 10 times” (4)). Cronbach’s alpha = .92.

After completing the training task, participants reported how many standard drinks they intended to drink each day of the following week (adapted from the alcohol consumption measure) [29]. Cronbach’s alpha = .65.

Current alcohol cravings were assessed via the 12-item Alcohol Craving Questionnaire—Short Form—Revised questionnaire (ACQ) [32]. Participants reported their agreement with statements such as “I miss drinking” and “If I used alcohol, I would feel less tense” on a 7-point scale anchored by “strongly disagree” and “strongly agree.” Cronbach’s alpha = .86.

Participants rated the appeal of each stimulus used in the AAT training task using the following scale, 1 (“I don’t find this appealing”) to 7 (“I find this extremely appealing”) [7]. Average ratings were computed for the alcohol and non-alcohol stimuli. These items were included to evaluate whether training affected preferences for alcohol or non-alcohol related stimuli [22]. Cronbach’s alpha for the approach avoid training were .95 (alcohol), .97 (non-alcohol); for general identity were .97 (alcohol), .82 (non-alcohol); and for personalized identity were .91 (alcohol), .89 (non-alcohol).

#### Procedures

Procedures were approved by the University of Washington’s Institutional Review Board. Participants were recruited from a randomized list of approximately 2500 current, full-time undergraduate students between the ages of 18–25 obtained from the university registrar’s office and invited via email to participate in a study about cognitive processes and alcohol. Forty-four percent of the students contacted agreed to participate in an online screening. They were provided with a link to a website where volunteers indicated their agreement with an information statement in place of written informed consent and completed an online battery of questionnaires as part of the screening process. All participants were compensated $15 for completing the online survey. In addition, participants who reported at least one episode of heavy episodic drinking (HED) in the previous month (defined as 4 or more drinks on one occasion for women and 5 or more drinks for men) were invited to participate in the follow-up lab study. Approximately 58% of participants met these heavy episodic drinking criteria and were invited to participate in the follow-up study, which took place over two sessions in the lab.

Participants who met HED criteria were directed to a website which informed them about duration of and compensation for the lab studies. Interested students were able to schedule a lab session directly over the Internet at that time. Of the 638 participants eligible to participate in the lab sessions, 46% scheduled and attended the initial lab session. Participants were guided through written informed consent at the beginning of each lab session and completed all measures in a room, with up to four participants present. All individual computers were equipped with privacy screens and partitions.

At the start of the initial 45-minute session, participants were assigned to the general identity, personalized identity, or approach avoidance conditions, which subsequently determined the version of the IAT and AAT assessment and training they completed. Participants first completed a randomized battery and either an alcohol approach IAT (for participants in the approach avoidance condition) or drinking identity IAT (for participants in the general and personalized identity conditions). They were then randomly assigned to the control or experimental condition for the AAT assessment and training. Following completion of the AAT assessment and training, participants were able to schedule the second lab session. Participants who declined to return for a second session were debriefed at this time. All participants were compensated $20.

During the second 45-minute session, participants completed a self-reported alcohol consumption measure (the DDQ). Then they completed the same AAT assessment and training (in that order) that they experienced in the first session, followed by the self-reported measures about alcohol and other beverage preferences, cravings, and future drinking intentions, as well as the same IAT from session 1. All participants underwent a funneled debriefing procedure to check for awareness about whether they were assigned to experimental or sham training after conclusion of the session and received an additional $25 for completing the second lab session.

## Results and Discussion

### Baseline Drinking Behaviors

First, baseline differences in drinking behaviors (alcohol consumption, RAPI scores, or AUDIT scores) as a function of condition (experimental or control) were evaluated. Note that any differences should be due to chance as participants were randomly assigned to condition. Significant differences in AUDIT scores were found in the approach avoid training groups (see Table 1 for statistics for all baseline group comparisons), with higher scores in the experimental condition. No significant differences emerged between the experimental and control conditions for the standard identity or personalized identity training groups.

**Table 1:** Study 1 Baseline Drinking Variables as a Function of Training Type and Group.

	Experimental	Control	*t* score	*p* value	Effect size
Variable	M + SD	M + SD			Cohen’s *d*
	*Approach Avoid Training* (*N* = 54 experimental, 46 control)				
Drinks per week	11.21 + 10.66	8.74 + 7.30	1.32	0.19	0.27
Heavy drinking episodes	4.06 + 3.34	2.98 + 2.53	1.79	0.08	0.36
AUDIT score	10.35 + 6.40	7.61 + 5.12	2.34	0.02	0.47
Alcohol-related problems	8.69 + 11.64	7.04 + 10.30	0.74	0.46	0.15
	*General Identity Training* (*N* = 52 experimental, 41 control)				
Drinks per week	9.39 + 8.24	9.78 + 8.03	0.23	0.81	0.05
Heavy drinking episodes	3.55 + 2.94	3.61 + 2.78	0.10	0.92	0.02
AUDIT score	8.19 + 4.50	9.15 + 5.19	0.95	0.35	0.20
Alcohol-related problems	8.69 + 9.34	8.88 + 10.77	0.09	0.93	0.02
	*Personalized Identity Training* (*N* = 52, experimental, 50 control)				
Drinks per week	10.10 + 9.01	8.72 + 6.32	-0.86	0.39	.18
Heavy drinking episodes	3.69 + 2.83	3.58 + 2.99	-0.18	0.86	.04
AUDIT score	10.19 + 5.65	9.14 + 5.52	-0.90	0.37	.20
Alcohol-related problems	9.76 + 9.91	6.84 + 5.57	-1.78	0.08	.37

*Note*. AUDIT score = score on the Alcohol Use Disorders Identification Test, higher scores = higher risk of alcohol use disorders. Cohen’s *d* = 2*t* /(*df* ^5)

#### Analytic Approach to Test for Training Effects

Next, we conducted a series of analyses to test for training effects for each of the three training types. There was attrition (on average 24%) from session 1 to session 2: for approach avoid training, 13 experimental and 6 controls dropped out; general identity training: 15 experimental and 9 controls dropped out; and personalized identity training, 14 experimental and 16 controls dropped out. We elected to analyze the data only from those who completed both sessions. We made this choice because we wanted to maximize the likelihood of detecting training effects, and our primary question concerned whether completing training would be effective, which required participants receive an adequate dose of training. We tested whether there were differences in baseline drinking (alcohol consumption, RAPI scores, or AUDIT scores) or baseline implicit alcohol associations (measured using the AAT and IAT) and found no evidence of differences between completers and non-completers, all *p*s > .16.

For variables that were assessed at pre- and post-training (e.g., implicit alcohol associations, cravings), we conducted a series of 2 x 2 mixed measures ANOVAs (with experimental vs. control condition as the between-subjects factor; and pre-training vs. post-training time as the within-subjects factor). A training effect would be indicated by a significant condition x time interaction. For variables only assessed at post-training (e.g., preferences for AAT stimuli), a one-way ANOVA was used to test for differences as a function of condition.

Given the baseline group difference in AUDIT scores, we analyzed the data for the approach avoid group two ways: including AUDIT scores as a covariate or as a moderator of condition (experimental vs. control). We compared the results from both strategies, and the pattern was identical. We report analyses using the covariate approach for simplicity.

#### Effects of Training on Implicit Alcohol Associations

We first examined whether there were differences in implicit alcohol associations as a function of condition using mixed measures ANOVAs. For each training type, we tested for differences in the AAT bias assessment (including differences in overall bias, alcohol bias, and water bias) and in IAT scores. Contrary to predictions, there were no significant condition x time interactions (please see Table 2 for means, SDs, and statistics for all condition comparisons) for any of the three training types. One marginal interaction was observed: there was a trend toward an increase in the non-alcohol AAT bias for the experimental compared to control condition. Overall, however, there was no evidence that the cognitive bias training significantly affected implicit alcohol associations, whether measured by the AAT or the IAT.

**Table 2:** Means, Standard Deviations, and Tests for Training Effects for Experimental and Control Groups in Study 1.

	Experimental		Control			
	Pre-training	Post-training	Pre-training	Post-training	Training Effect	Effect Size
Variable	Mean + SD	Mean + SD	Mean + SD	Mean + SD	F, *p*-value	partial η^2^
	*Approach Avoid Training (n = 41 experimental*, *40 control)*					
AAT Overall Bias	0.02 + 0.23	-0.02 + 0.20	0.05 + 0.23	0.01 + 0.24	*F* = 0.02, *p* = .88	3.1 x 10^−4^
AAT Alcohol Bias	0.02 + 0.42	0.04 + 0.34	0.12 + 0.30	0.27 + 0.38	*F* = 0.60, *p* = .44	.01
AAT Non-alc Bias	-0.02 + 0.41	0.09 + 0.36	0.04 + 0.44	0.23 + 0.38	*F* = 0.36, *p* = .55	.01
Approach IAT	-0.18 + 0.39	-0.18 + 0.33	-0.40 + 0.38	-0.34 + 0.38	*F* = 0.51, *p* = .48	.01
Intentions	11.05 + 10.00	10.57 + 9.85	7.79 + 5.93	8.90 + 8.99	*F* = 1.40, *p* = .21	.02
Cravings	-12.79 + 10.15	-12.26 + 13.27	-15.82 + 10.13	-13.67 + 13.53	*F* = 2.50, *p* = .12	.03
Alcohol IP		2.60 + 1.32		2.38 + 1.39	*F* = 0.02, *p* = .88	2.8 x 10^−4^
Non-alc IP		5.33 + 1.43		5.51 + 1.21	*F* = 0.15, *p* = .70	1.9 x 10^−3^
	*General Identity Training (n = 37experimental*, *32 control)*					
AAT Overall Bias	0.05 + 0.29	-0.02 + 0.23	-0.08 + 0.24	-0.02 + 0.26	*F* = 2.26, *p* = .14	.03
AAT Alcohol Bias	0.16 + 0.43	0.11 + 0.40	0.02 + 0.35	0.18 + 0.47	*F* = 2.56, *p* = .11	.04
AAT Non-alc Bias	0.07 + 0.44	0.15 + 0.42	0.16 + 0.50	0.21 + 0.39	*F* = 0.04, *p* = .85	5.5 x 10^−3^
Identity IAT	0.12 + 0.35	0.07 + 0.41	0.17 + 0.27	0.15 + 0.40	*F* = 0.71, *p* = .40	.01
Intentions	11.00 + 0.23	12.51 + 11.91	8.42 + 6.65	8.52 + 9.82	*F* = 0.76, *p* = .39	.01
Cravings	-13.74 + 10.09	-16.08 + 10.82	-11.76 + 13.12	-12.12 + 12.55	*F* = 0.52, *p* = .47	7.4 x 10^−3^
Alcohol IP		2.69 + 1.22		2.77 + 1.40	*F* = 0.07, *p* = .80	9.4 x 10^−4^
Non-alc IP		5.37 + 0.75		5.23 + 0.66	*F* = 0.69, *p* = .41	.01
	*Personalized Identity Training (n = 38 experimental*, *34 control)*					
AAT Overall Bias	0.04 + 0.26	-0.01 + 0.24	-0.04 + 0.23	0.00 + 0.20	*F* = 0.08, *p* = .79	.02
AAT Alcohol Bias	0.14 + 0.42	0.19 + 0.42	0.13 + 0.38	0.16 + 0.43	*F* = 0.03, *p* = .87	4.1 x 10^−4^
AAT Non-alc Bias	0.05 + 0.35	0.22 + 0.42	0.21 + 0.42	0.18 + 0.38	*F* = 3.14, *p* = .08	.04
Identity IAT	0.23 + 0.41	0.17 + 0.34	0.29 + 0.38	0.23 + 0.37	*F* = 0.00, *p* = .95	6.1 x 10^−5^
Intentions	9.30 + 7.07	9.65 + 7.70	9.03 + 6.46	9.27 + 7.69	*F* = 0.01, *p* = .93	1.1 x 10^−4^
Cravings	-14.41 + 11.67	-14.95 + 12.61	-11.50 + 11.69	-11.12 + 11.77	*F* = 0.19, *p* = .66	2.7 x 10^−3^
Alcohol IP		3.42 + 1.13		3.60 + 1.13	*F* = 0.47, *p* = .49	0.01
Non-alc IP		5.68 + 0.63		5.66 + 0.58	*F* = 0.01, *p* = .93	1.3 x 10^−4^

*Note*. Training effect refers to the results of the ANOVAs used to test for possible training effects (i.e., the results of the condition x time interactions for all variables other than cravings, alcohol and non-alcohol image preferences; for those variables, the results refer to condition main effect). Analyses for approach avoid training include AUDIT scores as a covariate. Partial η^2^ = SS _effect_ / (SS _effect_ + SS _error_). Scores for the AAT and IAT are D scores with higher scores indicating greater bias. Intentions = future drinking intentions or number of drinks participants expect to drink over the next week. Alcohol and Non-alc IP = alcohol and non-alcohol image preferences or the ratings of the images used in the AAT, higher scores = image being more appealing.

#### Effects of Training on Alcohol Outcomes

Next we examined whether there were changes in other alcohol-related outcomes; specifically, future drinking intentions, current alcohol cravings, or preferences for the alcohol- or non-alcohol-related stimuli used in the training tasks. Contrary to predictions but consistent with the findings reported above, no significant condition x time interactions were found for drinking intentions or cravings for any of the training types, and there were no condition differences in stimuli preferences for any of the training types (see Table 2 for statistics for all condition comparisons).

### Summary of Study 1 Findings

There was no evidence of training effects in the study. Neither the implicit alcohol associations nor the alcohol-related outcomes were affected by training, and these null findings were consistent across three training types (i.e., alcohol approach, general identity, and personalized identity). These findings were not as predicted and are inconsistent with those from other studies [2,3,22].

When considering these null findings, a number of factors could be critical. First, the interval between the sessions (approximately one week) may have been too long, allowing the training effects from the first session to fade. Wiers et al.’s study [3], for example, included four sessions on four consecutive days. Second, it is possible that the sample’s drinking behaviors and cognitive biases were not severe enough. The mean number of self-reported drinks peer week in our US sample ranged from 8 to 11 depending on training type whereas the Dutch undergraduate sample in Wiers et al.’s study [22] self-reported an average of 14 to 18 standard drinks per week depending on condition (note that these values were converted to US standard drinks to enable direct comparison) and study eligibility criteria included an AUDIT score of 8 or more. Third, there were occasional reports from participants, across all training types, that the AAT training and bias assessments were onerous and slow.

## Overview of Study 2

A second study was, therefore, conducted, but with modifications to address each of these factors. First, the two lab sessions occurred during the same week to reduce the time between sessions; this change might have the additional benefit of reducing the attrition rate. Second, to maximize the possibility of detecting any changes in implicit bias within each lab-session (in case effects were fading between sessions), participants completed the AAT bias assessment twice during each lab-session—immediately before and after completing the AAT training task. Further, following the second administration of the bias assessment, participants completed a brief (80-trial) block of the training task to reinforce their learning. Thus, at each lab-session, the AAT included a bias assessment, training task, follow up bias assessment, and booster training block. Third, a heavier drinking sample was recruited (individuals with AUDIT scores of 8 or more—a score that indicates possible hazardous alcohol use [30]), thereby matching the criteria used by Wiers et al. [22]. Fourth, the inter-trial time on the AAT assessment and training was reduced from 800 to 300 milliseconds, an interval consistent with the inter-trial time of the IAT, given participant complaints about the slowness of the task. Finally, an additional outcome measure, a measure of one’s ability to refuse alcohol, was administered to test for novel training effects.

## Study 2

### Method

#### Participants

The 288 participants in this study (150 women, 138 men), were undergraduate students recruited from the same university as in Study 1. All participants were fluent in English, between the ages of 18 and 25 years old (*M* = 20.46, *SD* = 2.14), and scored an 8 or greater on the AUDIT. 66% of the participants identified themselves as White, 15% as Asian, 12% as multiracial, 3% as African American, 1% as American Indian/Alaska Native, 1% Native Hawaiian or other Pacific Islander and 3% of participants chose “unknown” or did not respond. 88% identified their ethnic background as not Hispanic or Latino, 8% as Hispanic or Latino, and 4% selected “unknown” or chose not to respond.

#### Measures and Materials

As in Study 1, the Alcohol AAT consisted of a bias assessment and a training task. The same three variants of the AAT were used in Study 2: the original IAT, the general identity AAT, and the personalized identity IAT. The procedures for the AAT bias assessment and training tasks were identical to the procedures used in Study 1 with two changes. First, we reduced the inter-trial time to 300 milliseconds. Second, after completing the three blocks of the training task, participants completed the bias assessment again (to test for any immediate changes in bias). The second bias assessment was immediately followed by an 80-trial block of the same training task to reinforce participants’ learning. Split half reliabilities were again calculated to assess internal consistencies, and were again very low (*r*s for overall AAT D score: original AAT = -.09, general identity AAT = .04, personalized identity AAT = -.25; alcohol-bias D score: original AAT = .12, general identity AAT = .21, personalized identity AAT = .014; non-alcohol bias D scores: original AAT = .21, general identity AAT = .26, personalized identity AAT = .00).

The same IATs (Approach-Avoid and Drinking Identity) that were administered in Study 1 were used in Study 2. As in Study1, participants completed the IAT that matched the training type to which they were assigned. Internal consistencies were calculated using the same strategy as in Study 1: *r*s = .43 (approach avoid), .49 (drinking identity).

In the online screening survey, the Daily Drinking Questionnaire (DDQ) [29] was again used to measure participants’ typical weekly alcohol consumption. Cronbach’s alpha = .64.

Participants reported the number of heavy episodic drinking incidents they had experienced in the previous 30 days.

The Alcohol Use Disorders Identification Test (AUDIT) [30] was used to identify participants with an elevated risk for alcohol use disorders (i.e., scores of 8 or greater). Cronbach’s alpha = .59.

The Rutgers Alcohol Problems Index (RAPI) [31] was used to assess how frequently participants experienced 25 alcohol-related problems in the past three months. Cronbach’s alpha = .90.

After completing the training task, participants reported the quantity of standard drinks that they intended to consume on each day of the following week [29]. Cronbach’s alpha = .66.

The Alcohol Cravings Questionnaire (ACQ) was again used to assess current craving for alcohol (ACQ) [32]. Cronbach’s alpha = .81.

Participants rated their preferences for the stimuli used in the AAT training task using the same approach as in Study 1. Cronbach’s alphas for approach avoid training were .94 (alcohol), .97 (non-alcohol); for general identity were .96 (alcohol), .85 (non-alcohol); and for personalized identity were .94 (alcohol), .80 (non-alcohol).

The Drinking Refusal Self-Efficacy Questionnaire—Revised in an Adolescent Sample (DRSEQ) [33] was used to measure participants’ belief in their ability to refuse alcohol in 19 situations. Participants rated their confidence in their ability to resist alcohol in each of these situations on a six-point scale ranging from 1 (“I am very sure I could NOT resist drinking”) to 6 (“I am very sure I could resist drinking”). The items form three factors: social pressure self-efficacy (sample item: “when someone offers me a drink”), opportunistic self-efficacy (sample item: “when I am watching TV”), and emotional relief self-efficacy (sample item: “when I feel upset”). Cronbach’s alphas = .88 (social pressure), .85 (opportunistic), .94 (emotional relief).

#### Procedures

Participants completed an online screening questionnaire and those who met the revised eligibility criteria of AUDIT scores greater than or equal to 8 were invited to participate in two lab-based follow-up sessions that would occur within the same week. The remaining procedures for Study 2 were unchanged from those in Study 1.

### Results and Discussion

#### Baseline Drinking Behaviors

We again began by testing for baseline differences in drinking characteristics (alcohol consumption, RAPI scores, or AUDIT scores) as a function of condition (experimental or control). See Table 3 for means, SDs, and statistics for all baseline condition comparisons. A significant difference in AUDIT scores was found in the approach avoid training group, with significantly higher scores in the experimental condition. There was a marginal effect for a condition difference in heavy episodic drinking for the standard identity group, with marginally more drinking in the control condition. Finally, the experimental condition in the personalized identity group reported consuming significantly more drinks per week than the control condition.

**Table 3:** Study 2 Baseline Drinking Variables as a Function of Training Type and Group.

	Experimental	Control	*t* score	*p* value	Effect size
Variable	M + SD	M + SD			Cohen’s *d*
	*Approach Avoid Training* (*N* = 47 experimental, 43 control)				
Drinks per week	16.23 + 12.87	14.05 + 9.03	-0.94	0.35	.20
Heavy drinking episodes	4.87 + 3.47	4.53 + 2.95	-0.50	0.62	.11
AUDIT score	13.74 + 4.78	11.56 + 4.58	-2.48	0.02	.52
Alcohol-related problems	13.09 + 12.41	9.07 + 7.00	-1.91	0.06	.40
	*General Identity Training* (*N* = 50 experimental, 41 control)				
Drinks per week	12.35 + 8.03	14.76 + 8.31	1.40	0.17	.30
Heavy drinking episodes	4.32 + 2.85	5.41 + 2.65	1.89	0.06	.40.
AUDIT score	11.24 + 3.53	12.05 + 3.31	1.12	0.27	.24
Alcohol-related problems	11.00 + 12.16	10.37 + 9.87	-0.27	0.79	.06
	*Personalized Identity Training* (*N* = 55, experimental, 46 control)				
Drinks per week	17.15 + 11.07	12.63 + 7.99	-2.31	0.02	.46
Heavy drinking episodes	5.82 + 3.19	4.74 + 3.08	-1.72	0.08	.35
AUDIT score	12.69 + 3.85	11.89 + 4.58	-0.95	0.34	.20
Alcohol-related problems	11.02 + 9.38	12.28 + 11.37	0.61	0.54	.12

*Note*. AUDIT score = score on the Alcohol Use Disorders Identification Test, higher scores = higher risk of alcohol use disorders. Cohen’s *d* = 2*t* /(*df* ^5).

#### Analytic Approach

We used the same approach to test for training effects for each of the three training variants. There was attrition from session 1 to session 2 (18%, on average): for approach avoid training, 5 experimental and 1 controls dropped out; for general identity training: 9 experimental and 6 controls dropped out; and for personalized identity training, 13 experimental and 7 controls dropped out. We again analyzed data only from those who completed both sessions. We again tested whether there was evidence of differences in baseline drinking (alcohol consumption, RAPI scores, or AUDIT scores) and baseline implicit alcohol associations (on the AAT and IAT) between completers and non-completers. There were no significant differences between completers and non-completers in the personalized identity training group, all *p*s > .17); minor differences in the alcohol approach training group (i.e., non-completers had lower levels of heavy episodic drinking and lower AAT alcohol bias scores) and the general identity training group (i.e., non-completers had stronger AAT non-alcohol bias scores).

Given the significant baseline differences in AUDIT scores for the approach avoid training group and in drinks per week for the personalized identity group, we again ran the analyses two ways: including the AUDIT and drinks per week as covariates or as moderators for the relevant training type analyses. There were no differences in the pattern of results, so we again report analyses using the covariate approach for simplicity.

#### Effects of Training on Implicit Alcohol Associations

A series of 2 x 2 mixed measures ANOVAs were again used to test for differences in implicit associations as a function of training, with the significant baseline drinking variables included as covariates. See Table 4 for means, SDs, and statistics for all condition comparisons. Contrary to expectations but consistent with Study 1, we again found no evidence of training effects on AAT bias, including overall bias, alcohol bias and non-alcohol bias. A marginal interaction on the alcohol bias AAT score was observed for the approach avoid training group; the direction of the interaction indicated a trend toward decreasing alcohol bias in the experimental condition and increasing alcohol bias in the control condition. There was no evidence of training effects on IAT scores for any training variant.

**Table 4:** Means, Standard Deviations, and Tests for Training Effects for Experimental and Control Groups in Study 2.

	Experimental		Control			
	Pre-training	Post-training	Pre-training	Post-training	Training Effect	Effect Size
Variable	Mean + SD	Mean + SD	Mean + SD	Mean + SD	F, *p*-value	partial η^2^
	*Approach Avoid Training (n = 42 experimental*, *42 control)*					
AAT Overall Bias	0.01 + 0.17	-0.07 + 0.22	-0.01 + 0.25	0.01 + 0.28	*F* = 1.55, *p* = .22	.02
AAT Alcohol Bias	0.09 + 0.31	0.01 + 0.41	-0.06 + 0.32	0.02 + 0.47	*F* = 3.52, *p* = .07	.05
AAT Non-alc Bias	0.12 + 0.46	0.15 + 0.38	-0.05 + 0.44	0.01 + 0.39	*F* = 0.04, *p* = .86	5.1 x 10^−4^
Approach IAT	-0.31 + 0.41	-0.31 + 0.30	-0.27 + 0.37	-0.28 + 0.33	*F* = 0.00, *p* = .96	2.8 x 10^−5^
Intentions	11.55 + 10.66	15.40 + 12.53	11.14 + 7.66	12.00 + 8.20	*F* = 2.89, *p* = .09	.04
Cravings	-12.93 + 14.62	-9.95 + 13.87	-14.63 + 11.43	-12.34 + 10.28	*F* = 0.17, *p* = .68	2.3 x 10^−3^
Social Pressure	13.74 + 5.07	4.02 + 5.29	15.89 + 6.28	16.17 + 5.52	*F* = 0.01, *p* = .94	8.3 x 10^−5^
Opportunistic	38.52 + 5.26	39.36 + 4.15	39.06 + 4.47	38.63 + 4.06	*F* = 0.64, *p* = .43	8.6 x 10^−3^
Emotional Relief	34.79 + 7.93	35.24 + 6.32	34.37 + 7.82	34.26 + 6.51	*F* = 0.11, *p* = .74	1.4 x 10^−3^
Alcohol IP		2.62 + 1.25		2.73 + 1.19	*F* = 0.63, *p* = .43	8.4 x 10^−3^
Non-alc IP		5.38 + 1.35		5.04 + 1.61	*F* = 0.85, *p* = .36	.01
	*General Identity Training (n = 41 experimental*,* 35 control)*					
AAT Overall Bias	0.02 + 0.21	-0.04 + 0.24	0.04 + 0.23	0.01 + 0.24	*F* = 0.12, p = .73	1.6 x 10^−3^
AAT Alcohol Bias	0.09 + 0.40	0.04 + 0.42	0.14 + 0.41	0.11 + 0.57	*F* = 0.03, p = .86	4.1 x 10^−4^
AAT Non-alc Bias	0.05 + 0.41	0.14 + 0.44	0.08 + 0.33	0.11 + 0.46	*F* = 0.60, p = .44	.01
Identity IAT	0.29 + 0.34	0.30 + 0.37	0.40 + 0.44	0.29 + 0.36	*F* = 2.95, p = .09	.04
Intentions	10.95 + 6.18	13.24 + 8.70	12.77 + 8.76	14.17 + 11.45	*F* = 0.40, p = .53	5.4 x 10^−3^
Cravings	-13.61 + 10.01	-11.66 + 10.24	-15.46 + 11.65	-14.37 + 11.53	*F* = 0.19, *p* = .67	2.5 x 10^−3^
Social Pressure	14.66 + 5.86	14.46 + 4.96	16.94 + 6.04	14.74 + 5.24	*F* = 5.04, *p* = .03	.06
Opportunistic	38.85 + 4.43	38.83 + 4.09	40.63 + 3.05	39.11 + 7.54	*F* = 4.26, *p* = .04	.05
Emotional Relief	35.61 + 6.68	35.63 + 6.56	37.11 + 6.87	34.71 + 7.54	*F* = 6.48, *p* = .01	.08
Alcohol IP		3.40 + 1.34		2.93 + 1.27	*F* = 2.50, *p* = .28	.03
Non-alc IP		5.38 + 0.66		5.28 + 0.85	*F* = 0.30, *p* = .59	4.0 x 10^−3^
	*Personalized Identity Training (n = 42 experimental*, *39 control)*					
AAT Overall Bias	0.06 + 0.17	0.01 + 0.22	0.04 + 0.26	0.03 + 0.22	*F* = 0.40, *p* = .53	5.0 x 10^−3^
AAT Alcohol Bias	0.24 + 0.32	0.14 + 0.39	0.23 + 0.31	0.13 + 0.39	*F* = 0.02, *p* = .89	2.2 x 10^−4^
AAT Non-alc Bias	0.19 + 0.34	0.11 + 0.41	0.14 + 0.47	0.09 + 0.39	*F* = 0.06, *p* = .80	7.9 x 10^−4^
Identity IAT	0.30 + 0.35	0.17 + 0.32	0.39 + 0.36	0.33 + 0.30	*F* = 0.72, *p* = .40	.01
Intentions	13.60 + 12.02	15.90 + 10.69	10.77 + 7.53	14.38 + 16.19	*F* = 0.15, *p* = .70	1.8 x 10^−3^
Cravings	-14.43 + 13.41	-13.10 + 11.11	-15.23 + 10.91	-11.77 + 9.96	*F* = 0.78, *p* = .38	.01
Social Pressure	17.12 + 5.37	14.98 + 4.82	15.97 + 6.59	15.33 + 5.06	*F* = 3.46, *p* = .07	.04
Opportunistic	39.19 + 5.20	38.76 + 3.99	40.10 + 3.02	39.62 + 2.93	*F* = 0.00, *p* = .97	2.3 x 10^−4^
Emotional Relief	35.29 + 8.37	35.07 + 7.38	36.13 + 7.02	35.26 + 5.68	*F* = 0.14, *p* = .71	1.7 x 10^−3^
Alcohol IP		4.06 + 0.89		4.12 + 1.09	*F* = 1.55, *p* = .22	.02
Non-alc IP		5.73 + 0.48		5.78 + 0.63	*F* = 0.14, *p* = .71	1.8 x 10^−3^

*Note*. Training effect refers to the results of the ANOVAs used to test for possible training effects (i.e., the results of the condition x time interactions for all variables other than cravings, alcohol and non-alcohol image preferences; for those variables, the results refer to condition main effect). Analyses for approach avoid training include AUDIT scores as a covariate; analyses for personalized identity include drinks per week as a covariate. Partial η^2^ = SS _effect_ / (SS _effect_ + SS _error_). Scores for the AAT and IAT are D scores with higher scores indicating greater bias. The AAT bias scores presented here are the baseline bias scores and the bias scores immediately following the second training session. Intentions = future drinking intentions or number of drinks participants expect to drink over the next week. Social Pressure, Opportunistic, Emotional Relief = drinking-related self-efficacy factors with higher scores indicating greater self-efficacy. Alcohol and Non-alc IP = alcohol and non-alcohol image preferences or the ratings of the images used in the AAT, higher scores = image being more appealing.

Because we included a bias assessment immediately following each training session, we were able to evaluate potential changes in AAT bias at each session. Thus, we conducted exploratory analyses that tested for changes within each session as well as across all four bias assessments. Those were conducted for the overall, alcohol, and non-alcohol bias scores. There was no evidence of training effects for the general identity or personalized identity AAT bias scores, all *p*s > .05. One significant difference was found for the approach avoid training group: when evaluating change in alcohol bias within the first training session, there was a significant interaction, with alcohol bias decreasing for the experimental group and increasing for the control group, *p* = .003.

#### Effects of Training on Alcohol Outcomes

Finally, we tested for changes in other alcohol-related outcomes as a function of training group. See Table 4 for means, SDs, and statistics for all condition comparisons. There were no significant training effects on future drinking intentions, craving, or preferences for the AAT stimuli. A marginal interaction effect on future drinking intentions was observed for the approach avoid training group; the direction of the interaction indicated marginally larger increases in future drinking intentions for those in the experimental compared to those in the control condition.

Drinking refusal self-efficacy yielded a somewhat different pattern. There were no training effects on drinking refusal self-efficacy for the approach avoid training group, but significant interactions were observed for all three drinking refusal self-efficacy variables for the general identity training group. However, the pattern of those differences indicated no changes in drinking self-refusal for the experimental conditional, and reductions in self-efficacy for the control group. Thus, the observed differences were not consistent with expectations. A marginal effect of training was observed in the personalized identity training group for one of the three drinking refusal self-efficacy variables (e.g., social pressure). The pattern of results indicated a trend toward a reduction in self-efficacy among the experimental condition but not the control condition. Thus, it also was not consistent with expectations.

### Summary of Study 2 Findings

Study 2 was conducted to address possible factors that could have accounted for the lack of training effects in Study 1. Most importantly, we recruited a higher risk sample by requiring participants to have an AUDIT score of 8 or more, thereby matching the criteria used in Wiers et al. [22]. Study 2 participants had, on average, nearly equivalent AUDIT scores to Wiers et al. (in both studies, means ranged from 12 to 14) and reported similar mean levels of alcohol consumption (12 to 17 standard drinks per week in our study vs. 14 to 18 standard drinks per week in Wiers et al.). Follow-up analyses that compared Study 1 and Study 2 participants’ baseline drinking behaviors (drinks per week, heavy drinking episode, AUDIT scores, and alcohol-related problems) also provide evidence that the Study 2 sample was higher risk: study 2 participants’ baseline drinking behaviors were consistently and significantly higher than Study 1 participants, *p*s < .001. However, despite testing the training paradigms with higher risk participants and modifying procedures to maximize the possibility of detecting training effects (i.e., by reducing the time period between sessions, evaluating implicit bias immediately after completing the training task, and reducing the inter-trial time of the AAT), we again found no consistent evidence of training effects. That is, none of the three types of training—approach avoid, general identity, or personalized identity—yielded significant differences in implicit alcohol associations. We did observe one marginal difference in the expected direction for the alcohol bias AAT score, but that was the only (and admittedly slim) evidence of training effects. Occasional significant differences were observed on the drinking behaviors—specifically on the drinking refusal self-efficacy variable in the identity training types. Those differences, however, were not in the expected direction, and thus did not support the efficacy of training. No other significant differences were observed in the alcohol-related outcomes for any of the training types. Ultimately, Study 2 findings did not provide evidence for the efficacy of any of the training types, even in a higher risk sample.

## General Discussion

Contrary to hypotheses and previous findings, neither of our two studies found evidence for the efficacy of training implicit alcohol associations in US undergraduate drinkers. Three variants of the AAT were evaluated—the alcohol approach AAT training used successfully in Northern European samples [2,3,22], as well as two newly developed adaptations of the AAT that attempted to target implicit drinking identity associations—the standard and personalized identity AAT. Across two studies, there was no evidence that training shifted participants’ implicit alcohol associations. We also found no consistent evidence of change in any of the other outcomes (i.e., intentions, craving, preferences for alcoholic or non-alcohol stimuli, or drinking refusal self-efficacy). This lack of change is not surprising given the lack of shift in implicit associations (i.e., the lack of change in bias; see [34], for a similar argument regarding null findings in attentional bias training). Finally, we conducted post-hoc exploratory analyses that evaluated whether participant gender, independently or interactively, influenced the results, and we observed null findings for nearly all outcomes in both studies and across all three variants of the AAT training.

With respect to methodology, our studies used the AAT training developed by Wiers et al. [3] and the training condition that over-trained pushing alcohol away, but our studies did vary in several respects. Our studies included a sham training (control) condition (developed in Wiers et al.’s later studies, see 2, 3) instead of a second condition that over-trained pulling alcohol close. We also increased the number of trials for each training session, essentially doubling the training dose. Our studies also did not include a lab-based measure such as an alcohol taste test to evaluate immediate behavior changes in drinking. We suspect that even if we had included a taste test we would not have observed behavioral differences in a taste test given the lack of change in implicit associations. Finally, we attempted to create variants of the AAT that could be used to target drinking identity by utilizing words representing non-drinking parts of identity and adding the words “Me” and “Not Me” to the screen to clarify the relationship been zooming away/close and the self. We should note that while we found no significant differences in implicit associations in either study, occasional marginal training effects were found for approach avoid associations for those who completed the alcohol approach training (vs. sham training). Those effects could be spurious, but they also could be indicators of the potential for the original alcohol approach training effectiveness under the right conditions. In contrast, there was no evidence that either of the drinking identity training variants resulted in more adaptive implicit associations or drinking outcomes.

Unfortunately, we do not have clear evidence about which factor(s) are responsible for our null findings. However, our research team consists of researchers who have conducted multiple cognitive training studies targeting different maladaptive behaviors and mental health concerns, some of which have been successful [2,3,35–37] and some of which have not [38–40]. Accordingly, we offer some discussion of our findings and lessons learned, and provide recommendations for future studies. Our hope is that such a discussion may prove useful to others because training studies are resource-intensive.

## Implications and Lessons Learned

### Changes in Methodology and Participant Samples Did Not Yield Evidence of Training Effects

A critical lesson learned from the present studies was that methodology and participant changes did not lead to increased evidence of training effects. Our hope was that reducing the interval between training sessions, including an additional AAT bias assessment, reducing the inter-trial time on the AAT including an additional outcome measures, and recruiting a heavier (and more hazardous) drinking sample would increase the likelihood of detecting training effects. We found no evidence that these modifications increased the effectiveness of training. Because these modifications were implemented simultaneously in Study 2, note that we cannot, unfortunately, speak definitively about the (non-)effectiveness of any single modification.

A second lesson learned was that the adaptations of the AAT to target drinking identity were not effective. The design of the present studies precludes us from firm conclusions but two possibilities seem likely. First, it may be that identity associations, because they concern the self, are simply harder to shift. Second, it may be that displaying the word “not me” on the screen was a problem because “not me” still contains the word “me.” It may have made the task too difficult for participants and affected their results. Future studies might benefit from using words such as “others” or “someone else.”

### Training May Not Be Helpful for Non-treatment Seeking Undergraduates

While training implicit alcohol tendencies (specifically, implicit approach bias) with individuals with substance use disorders has proven to be a promising intervention for in-patient substance use treatment seekers [2,3], training, in its current form, may not serve a protective function in a non-treatment seeking undergraduate population. This is particularly unfortunate because undergraduates are in a crucial developmental stage in relation to substance use behavior [41]. Many young adults develop drinking habits during their college years that increase risk for substance use disorder later in life [41]. Further, although many students will “mature out” of the heavy drinking that occurs during the college years [8], there is a rapid cumulative increase in onset of heavy drinking from late teens through early adulthood [42]. Second, this group is frequently not interested in behavior change, and typically difficult to treat [43], which makes the current null findings particularly disappointing (i.e., training does not appear to represent a more interesting and/or effective alternative).

### Training May Be More Effective for Individuals With More Severe Drinking Histories and Stronger Cognitive Biases

A third implication of our null findings is that the population used to test for potential training effects is critical. Our rationale for selecting this particular group—US undergraduate drinkers—was that they are a group that tends to drink heavily and that their drinking is associated with substantial personal and societal costs [8,44]. We also hypothesized that their relative youth and shorter drinking histories might make them particularly responsive to training. However, our findings did not support the effectiveness of training in these participants. Given the positive findings from Europe, particularly in patients receiving treatment for alcohol dependence, it may be that our premise was faulty. That is, it may be that training, at least using the AAT, is more effective in individuals who are older, have longer drinking histories, experience more (and greater) alcohol-related negative consequences, and who, ultimately have stronger implicit alcohol association (stronger alcohol biases). In essence, these null results could be a function of restricted range in alcohol bias. This would be consistent with (1) the fact that participants in several training studies appeared to have higher scores on the alcohol approach IAT [3,22] than the participants in the current studies and (2) findings from Eberl et al. [2] of greater training effects in those who had a stronger bias (though this is not a consistent finding, especially in other disorder domains; e.g., anxiety-linked biases: [45] vs. [46]). Thus, future studies might benefit from targeting individuals with more severe drinking histories and stronger implicit alcohol associations (and ideally using more reliable measures of alcohol associations that may make it easier to detect change, given the especially low internal consistency of the AAT).

### Motivation to Train and Change May Be Critical for Successful Training

Underlying the implications discussed above is the likelihood that motivation to train [47] and/or to change is a critical element for successful cognitive training. Our participants were not necessarily motivated to train their associations or change their drinking. These studies were not clinical trials, were not described to participants as intervention studies, and did not specifically target individuals interested in changing their drinking. When originally designing these studies, our premise was that motivation to engage in training and/or change drinking was not critical: we were targeting associations presumed to be implicit (i.e., faster/impulsive/reflexive) and perhaps those associations and resulting behavior could be shifted without an express desire to change. Moreover, some of us had concerns that recruiting based on such desires could result in demand characteristics or placebo effects. The decisions not to recruit based on those characteristics and not to describe the study as a potential intervention may have undermined our attempt to demonstrate any training effects. In support of this possibility, many participants made it clear that the training tasks were tedious (this held true even after we reduced inter-trial times in Study 2). The internal consistencies of the AATs (and even the IAT) were low, which might be an indication of a lack of engagement in the training task as well as related computer-based reaction time tasks. Second, even if participants were more motivated or engaged in the training tasks, a more recent study on smoking found that that motivation to change the behavior (i.e., reduce smoking) is a critical element of successful training and behavior change [48]. Thus, future studies that seek to evaluate the efficacy of alcohol training in college samples might consider focusing specifically on high risk drinkers who are interested in reducing their drinking.

### Greater Doses May Be Necessary for Successful Training

Another implication of our null findings concerns dose of treatment, both in terms of the interval between doses and the number of doses. Our results suggest that two doses of training, even when decreasing the interval between them (from once a week to twice a week) are not enough to reduce implicit alcohol associations, let alone affect drinking outcomes. It may simply be too much to ask of these training tasks. Such reasoning is consistent with findings from Eberl and colleagues [49] that there are considerable individual differences in response to training using the AAT (ranging from five to 12 sessions), and there are no clear answers yet about what factors might identify those individuals who need more or less sessions. Some caution, however, is clearly indicated in terms of this recommendation given there is some evidence that one or two sessions of training can reduce implicit alcohol associations in Dutch college students [22] and German in-patient alcoholics [49]–i.e., it is unclear from our data whether the issue is that the bias is not strong enough, the dose is insufficient, or training sessions were spaced too far apart. It should be noted, however, the training studies showing robust effects were investigating training paired with other treatments for reducing drinking, such as an inpatient treatment program [3,49]. It is currently unclear if and when alcohol training can stand alone [38].

### Training May Be More Effective With Gamification and Motivational Enhancement

A final implication concerns the possibility of needing to change the training paradigm itself. Although not directly measured in this study, participants regularly reported lack of interest and engagement with the training tasks (e.g., “This is awful,” “Will I have to do it at the next session?”). Note that these complaints also occurred in Study 2 where we had reduced the inter-trial time on the AAT assessment and training in hopes of addressing concerns from Study 1 participants, which suggests that this reduction was not sufficient to increase interest or engagement. Further, attrition was high (i.e., 24% at Study 1 and 18% at Study 2). While this is a problem for any treatment protocol, this is particularly troubling given the characteristics of this population. Treatment effectiveness research for substance users has consistently found that client motivation and engagement play a crucial role in outcomes [50], with low motivation strongly predicting negative treatment outcomes. Within the field of treatment for substance use disorders, attendance [51], motivation [52,53], and engagement [54] have all been indicated as important predictors of treatment outcomes. While the AAT training task has been shown to be an effective intervention paradigm in individuals already seeking treatment for alcohol dependence, participants who lack motivation to change may be unlikely to benefit from the program. Other recent null findings in an online comparison of alcohol cognitive bias modification and placebo-training for problem drinkers by Wiers et al. [38] may be understood as an indication of the importance of the inclusion of a motivational enhancement component in training programs for individuals in a pre-contemplative stage of change. Future evaluations of alcohol approach bias retraining would likely benefit from the inclusion of a brief motivational enhancement intervention for individuals who may be more ambivalent about changing substance use behavior [55,56]. Additionally, improving the motivation and engagement of participants might be accomplished by enhanced gamification of the training procedure itself [57,58].

## Conclusion

This paper describes the results of two studies testing cognitive training on implicit alcohol associations in samples of US undergraduate drinkers. Across two studies, there was no consistent evidence that training decreased alcohol associations (whether alcohol approach or drinking identity) or affected other alcohol outcomes. We suggest that future training studies might benefit from focusing on heavier drinking individuals with stronger biases and who want to reduce their drinking, using more training sessions, pairing training with motivational enhancement, and increased gamification of the training task itself. While our findings were not in the expected direction, we hope that they and the lessons we have learned can be helpful to other researchers.
